# Uncovering core stemness biomarkers in HBV-HCC through integrated multi-omics

**DOI:** 10.1371/journal.pone.0349171

**Published:** 2026-05-14

**Authors:** Hanning Xu, Huan Li, Ling Hu, Xiaomei Wang

**Affiliations:** 1 Clinical Research Center of Gastroenterology Department, Tianyou Hospital, Affiliated to College of Medicine, Wuhan University of Science and Technology, Wuhan, Hubei; 2 Institute of Infection, Immunology and Tumor Microenvironment, School of Medicine, Wuhan University of Science and Technology, Wuhan, Hubei, China; Rutgers: Rutgers The State University of New Jersey, UNITED STATES OF AMERICA

## Abstract

Chronic hepatitis B virus (HBV) infection is a major etiology of hepatocellular carcinoma (HCC), with cancer stem cells (CSCs) playing a critical role in tumor progression and therapeutic resistance. However, the specific mechanisms linking HBV infection to stemness features in HBV-associated HCC (HBV-HCC) remain incompletely defined. To identify potential regulators, we conducted an integrated *in silico* multi-omics analysis using single-cell and bulk transcriptomic datasets. Comparative stemness scoring across major cell populations prioritized the endothelial compartment for further analysis. Subsequent pseudotime trajectory and CytoTRACE2 analysis of single-cell RNA sequencing (scRNA-seq) data identified Cluster 4 (C4) as a putative stemness-associated endothelial subpopulation, from which 21 differentially positive-HBV-expressed genes (DPEGs) were derived. In bulk RNA sequencing (bulk RNA-seq), weighted gene co-expression network analysis (WGCNA) identified genes strongly correlated with stemness in the blue module. Cross-referencing scRNA-seq and bulk RNA-seq yielded 52 shared genes. Random Forest feature selection prioritized two high-confidence biomarkers: *SSR2* and *UBE2D3*. A combined diagnostic model based on these two genes showed favorable performance across independent cohorts with bootstrap-supported validation, and survival analyses indicated that high *SSR2* expression and low *UBE2D3* expression were associated with poor clinical outcomes. Mechanistically, pathway activity correlation analyses revealed that *SSR2* and *UBE2D3* are significantly associated with endoplasmic reticulum stress (ERS), and mTOR signaling, suggesting a potential coordinated role in regulating viral persistence and stemness maintenance. Overall, our findings propose *SSR2* and *UBE2D3* as candidate diagnostic and prognostic biomarkers, providing a hypothesis-generating framework for developing targeted therapeutic strategies against HBV-HCC.

## Introduction

According to 2022 global statistics, liver cancer ranks as the sixth most common malignancy globally, with hepatocellular carcinoma (HCC) being the predominant pathological type [1,2]. HCC pathogenesis primarily stems from chronic liver injury. Chronic hepatitis B virus (HBV) elevates carcinoma risk through viral proteins and gene integration-induced chromosomal aberrations in host hotspots, leading to key oncogenic alterations [3,4]. Despite advances in understanding HCC pathogenesis, tumor recurrence and treatment resistance remain clinical challenges [5].

Cancer stem cells (CSCs) have been widely implicated in tumor initiation, recurrence, and drug resistance through their self-renewal and proliferative capacities. Furthermore, their differentiation potential significantly contributes to intra-tumoral heterogeneity [6]. Additionally, CSCs promote tumor progression through various biological processes, including immune evasion via MHC-I downregulation and endothelial transdifferentiation under hypoxic conditions [7]. Understanding the mechanistic interplay between CSCs and HCC is crucial for addressing the persistent challenges in HCC management. Multiple intrinsic regulators, including transcription factors (e.g., SOX4), non-coding RNAs (miR-21, lncRNA-HOTAIR), and epigenetic modifiers (EZH2, DNMT3A), converge on canonical signaling pathways to drive dysregulated self-renewal and enhance tumor-initiating potential in HCC [8–10].

A critical link between HBV, CSCs, and HCC pathogenesis is increasingly recognized. For instance, the HCC-related HBV protein HBx has been shown to enhance stemness-associated gene expression (e.g., EpCAM, Oct-4) through multiple pathways [11–13]. Furthermore, the aberrant activation of the mTOR signaling pathway has been extensively implicated in HBV-associated HCC (HBV-HCC) and liver CSCs (LCSCs) regulation, profoundly influencing tumor metabolism, immune evasion, and stemness maintenance [14–17]. However, the precise mechanism by which HBV mediates HCC development through regulating CSC biology remains elusive.

Advances in bioinformatics have significantly facilitated the identification of potential stem-related drivers for HBV-associated HCC progression. While bulk RNA-seq has been valuable for transcriptome profiling, its limited resolution of cellular heterogeneity has driven the adoption of scRNA-seq as a transformative technology. The high resolution of scRNA-seq allows for the identification of tumor-specific signatures and dissection of CSC heterogeneity at the single-cell level. For example, scRNA-seq has revealed that EpCAM + AFP+ subtypes and hepatic stem progenitor cell markers (CD133, CD44, SHP2) are associated with tumor aggressiveness and poor prognosis [18–20]. This technology has also facilitated the delineation of the tumor immune microenvironment, uncovering, for instance, reciprocal activation between CSCs and tumor-associated macrophages (TAMs) [21], and dynamic immune atlas mapping has provided mechanistic insights for combinatorial immunotherapy [22]. Integrating scRNA-seq with bulk RNA-seq leverages their complementary strengths: the latter offers a cost-effective transcriptomic overview of large cohorts, while the former resolves rare cell states and cell-cell interactions. Such integrative multi-omics approaches could systematically decode tumor ecosystems, bridging population-level molecular patterns with single-cell functional hierarchies to achieve a multidimensional understanding of HCC pathogenesis [23].

In summary, as CSCs are considered the cellular origin of many tumors, we hypothesized that HBV promotes the emergence and self-renewal of LCSCs through specific molecular mechanisms, thereby increasing susceptibility to HCC [24]. In this study, we employed an integrative *in silico* approach combining scRNA-seq and bulk RNA-seq to identify candidate stemness-associated genes in HBV-HCC, establishing a computational foundation for elucidating the pathogenic mechanisms underlying HCC and providing targets for future experimental validation.

## Materials and methods

### Data collection and processing

ScRNA-seq data of 3 HBV-positive (HBV status as annotated) HCC samples and 3 HBV-negative HCC samples from GSE282701 were downloaded from the Gene Expression Omnibus database (GEO, https://www.ncbi.nlm.nih.gov/geo/). For bulk transcriptomic analysis, two primary discovery cohorts were compiled: (i) MultiArray1, integrating datasets GSE121248 (HBV status as annotated) and GSE84402 (HBV status defined by seropositivity for Hepatitis B surface antigen [HBsAg]), comprising 83 HBV-positive HCC samples and 50 HBV-positive adjacent non-tumorous liver tissues. (ii) MultiArray2, integrating datasets GSE272035 (6 HBV(+) and 13 HBV(-) HCC) and GSE104310 (4 HBV(+) and 2 HBV(-) HCC) (HBV status for both as annotated in source databases). An independent validation cohort, GSE83148, comprising 122 HBV-positive HCC tissues and 6 HBV-negative normal liver tissues (HBV status defined by positive HBsAg or detectable serum HBV-DNA), was obtained from GEO to confirm expression patterns and diagnostic performance. Expression and clinical data for prognostic and pathway correlation analysis were sourced from 105 HBV-positive HCC patients (TCGA-LIHC-HBV(+)) within the Cancer Genome Atlas (TCGA, https://portal.gdc.cancer.gov/), where case inclusion required positive viral hepatitis serologies with at least a positive HBsAg test.

Stemness-related genes were identified based on Monocle2 using GSE282701 and on single-sample gene set enrichment analysis (ssGSEA) and weighted gene co-expression network analysis (WGCNA) using MultiArray1. Core stemness-associated genes were determined by integrated results (KEGG and Venn).

This core set was further used to identify core candidate genes by Random Forest (RF). HCC-associated (MultiArray1) and HBV-associated genes (MultiArray2) were separately identified. The intersection of HCC-associated and HBV-associated gene subsets identified candidate genes and further validation was carried out by expression levels.

**Fig 1:**
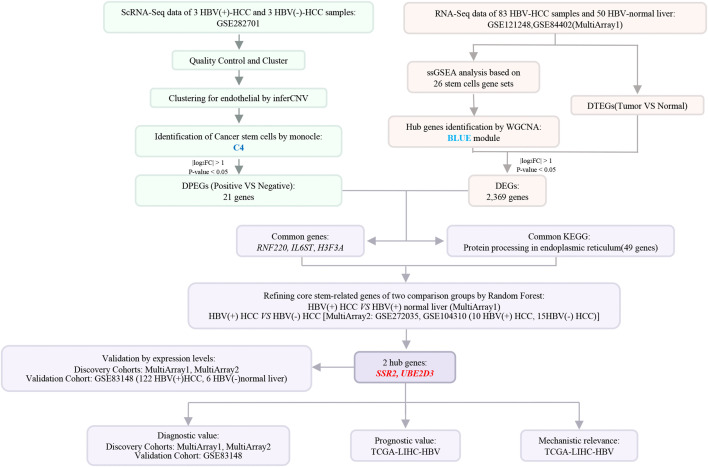
Graphical Abstract. HBV, hepatitis B virus; HCC, hepatocellular carcinoma; scRNA-seq, single-cell RNA sequencing; ssGSEA, single-sample gene set enrichment analysis; DPEGs, differentially positive-HBV-expressed genes; DEGs, tumor-associated stemness genes; DTEGs, differentially expressed tumor-enriched genes; WGCNA, weighted gene co-expression network analysis; KEGG, kyoto encyclopedia of genes and genomes.

The diagnostic and prognostic value were evaluated to establish clinical relevance. The overall workflow of the study is shown in Fig 1.

### Dimensionality reduction and clustering for scRNA-seq analysis

We used the Read10X function in Seurat package (https://CRAN.R-project.org/package=Seurat) to read the raw expression values of scRNA-seq data.

We used the PercentageFeatureSet function to calculate the percentage of mitochondrial genes (percent.mt) in each cell, and used FeatureScatter function to explore the relationship between nCount and nFeature, as well as the relationship between nCount and percent.mt. Cells with detected genes (nFeature_RNA) < 200 or > 4,500, total UMI counts (nCount_RNA) < 1,000 or > 25,000, and mitochondrial gene content (percent.mt) > 10% were removed. The strongly positive correlation between nCount_RNA and nFeature_RNA (Pearson r = 0.87); and the slight correlation between nCount_RNA and percent.mt (r = −0.09) demonstrated the efficiency of filtration.

We performed dimensionality reduction on preprocessed data. Data normalization was performed using the Normalize Data function to standardize the expression values of cells. Then we identified the top 2000 highly variable genes (HVGs) using the Find Variable Features function and used them for principal component analysis (PCA) in Seurat package. There might be some potential batch effects existing in the data that may confound the downstream analyses. We used the Harmony algorithm in Harmony R package to address these potential batch effects.

We performed non-linear dimensionality reduction using Uniform Manifold Approximation and Projection (UMAP) function to visualize cells in a low-dimensional space, and constructed a neighbor graph using Find Neighbors function and clustering using Find Clusters function. For cell type annotation, we used the top 5 marker genes for each cell cluster, identified using Find All Markers function with the Wilcoxon rank-sum test algorithm (min.pct = 0.25, logFC > 0.25). We annotated these markers using Cell Marker database (http://xteam.xbio.top/CellMarker/), and PanglaoDB (https://panglaodb.se/). We also cross-referenced these markers with well-known canonical markers for common cell types: Fibroblasts (*FGF7, MME, COL3A1, COL1A1*); Endothelial cells (*VWF, PECAM1, PLPP1*); Epithelial cells (*EPCAM, PROM1, ALDH1A1, CD24, KRT19, KRT18*); T cells (*CD3D, CD3E, TRAC, CD8A*); B cells (*MS4A1, CD79A, CD19*); NK cells (*FGFBP2, CX3CR1, KLRD1, GNLY, NKG7*); Monocytes (*CD68, CD163, CD14*); Mast cells (*KIT, TPSAB1, TPSB2*); Plasma cells (*MZB1, IGHG1, SDC1*). This approach defined eight major cell populations: T cells, Endothelial cells, Hepatocytes, Monocytes, Fibroblasts, NK cells, B cells, and Epithelial cells.

### Application of inferCNV and monocle in identifying CSCs subpopulations

We isolated endothelial cells based on an initial stemness potential screening. We calculated single-cell stemness scores for all major cell types using the AddModuleScore function in Seurat, referencing 9 stemness gene sets defined by gene expression, RNAi screens, transcription factor (TF) binding sites, literature reviews and computational approaches from StemChecker platform (http://stemchecker.sysbiolab.eu/). The endothelial cells exhibited a markedly heterogeneous and contextually elevated stemness potential compared to other cell types, warranting its selection for deeper investigation. We then performed copy number variation (CNV) analysis using inferCNV package (https://bioconductor.org/packages/infercnv/) on endothelial cluster. We partitioned the endothelial subpopulation into several clusters using K-means algorithm. UMAP was used for non-linear dimensionality reduction and visualization of endothelial cell heterogeneity. Pseudotemporal ordering of endothelial cells was performed using Monocle2 package (https://bioconductor.org/packages/monocle). DDRTree-based dimensionality reduction was used to reconstruct differentiation trajectories and order cells along pseudotime based on their transcriptional programs. To independently assess the stemness potential of endothelial subclusters, we calculated the CytoTRACE score for each cell using the CytoTRACE2 algorithm, which predicts cellular differentiation states based on gene expression diversity. Finally, we measured expression levels of CD34 (a canonical endothelial marker) and CD47 (a CSC marker) in all endothelial clusters.

### Differentially Positive-HBV-Expressed Genes (DPEGs)

To avoid introducing statistical pseudo-replication by mistakenly treating multiple cells from the same source as independent biological replicates, this study adopted a pseudo-bulk analysis strategy for all group comparisons based on single-cell data. We first aggregated the gene expression counts of all cells from the same patient sample in Cluster 4 to generate a representative expression profile of that sample within the subset. Subsequently, based on the aggregated sample-level expression matrix (HBV-positive group, n = 3 samples vs. HBV-negative group, n = 3 samples), differential expression analysis was performed using the limma R package. Significantly differentially expressed genes were identified with the following thresholds: absolute log2 fold change |log2FC| > 1 and P-value < 0.05.

### Bulk RNA-seq analysis of tumor and paracancer tumor-associated stemness genes (DEGs)

There were 2 HBV-negative samples in MultiArray1 (GSM2233104, GSM2233105). We excluded them here per the original experimental design. Using the GEOquery package, we read the corresponding matrix files for these data sets. The expression matrix and clinical information were read using exprs and pData functions, respectively.

Annotation information of their platform, GPL570, can be directly downloaded from GEOquery package. The probe IDs and Gene Symbols were extracted from the annotation information. Then use the merge function to make the gene IDs in the expression matrix match with the probe IDs from the microarray platform, which helps to convert gene names. Finally, use the limma package to compute the average value when one gene had several probes, and then finish the data forming. There may be some batch effects and heterogeneity between these two GSE datasets. Hence, we use the normalizeBetweenArrays function based on the limma package to normalize these two GSE datasets. After merging the two datasets, we use the removeBatchEffect function based on the limma package to remove the batch effects. Then use a microarray analysis method based on linear models using limma package to identify differences between tumor and non-tumor tissues.

The preprocessed gene expression data were fitted to a linear model using the lmFit function. Differential expression was compared between the tumor and the non-tumor. Bayesian statistical processing was conducted to make the statistical test more robust. We set the adjusted P-value threshold at 0.05 and the absolute value of log2 fold change (log2FC) at 1. We classified DEGs based on these criteria and marked them. At last, we drew a volcano plot for visualization.

### Integration and consensus clustering of HCC stemness signature

We downloaded 26 stemness gene sets from StemChecker platform, then imported into Excel and read into the R programming language. Based on the Gene Set Variation Analysis (GSVA) R package, we imported these 26 gene sets and performed the ssGSEA to score the enrichment levels of these 26 gene sets in samples (MultiArray1). Furthermore, we used the ggplot2 package to plot the expression levels of different gene sets in tumor tissues and adjacent non-tumor tissues. Finally, we used the t-test to compare the expression levels between two types of tissues and added the significance markers.

### WGCNA based on ssGSEA scoring

We used WGCNA package to construct co-expressed gene networks. Firstly, hierarchical clustering analysis was performed to detect potential outlier samples in MultiArray1. Then, we removed 32 outliers, and the remaining outlier-controlled datasets were used for network construction by threshold optimization. The co-expression similarity matrix was calculated based on Pearson’s correlation coefficients between all pairs of genes. Then, a weighted adjacency matrix was constructed by raising the similarity matrix to the power of β = 7, which could reflect a soft threshold and generate a scale-free topology. Next, the topological overlap matrix (TOM) was derived from the adjacency matrix and was used to cluster highly similar genes into modules. Module eigengenes (MEs) were calculated as the first principal component of each module expression matrix.

Finally, the association between modules and clinical features, including stemness scores (obtained by ssGSEA) and tumor/non-tumor classes, was evaluated by t-tests. The core gene modules significantly correlated with clinical features were visualized as heatmaps, and hub genes were extracted for downstream functional analysis.

### Gene function enrichment analyses and PPI construction

KEGG analysis was used to analyze the enrichment of differentially expressed genes from single-cell transcriptomics and bulk RNA-seq data in functional pathways, such as metabolic pathways, cellular signaling pathways, and regulatory pathways. Then, the target genes of similar pathways enriched in both single-cell and bulk RNA-seq data were screened out and used for PPI (Protein-protein interaction) analysis via the STRING database (https://www.string-db.org/) to identify central interacting genes.

### Selection of candidate diagnostic genes using random forest

To further screen for key candidate genes, the RF machine learning algorithm was applied to expression profiles of two comparison groups: (1) HBV(+) tumor tissue vs HBV(+) non-tumorous liver tissue (MultiArray1), and (2) HBV(+) HCC vs HBV(-) HCC (MultiArray2) using “randomForest” R package. This calculated a relative importance score for each gene. Then, the genes were ranked according to their relative importance scores, and the top genes with scores > 2 were selected. Candidate stem-related genes were subsequently identified by finding the intersection of genes obtained from RF models of HCC and HBV.

To validate our results, the “ggplot2” package was used to assess the expression levels of candidate genes between the control and disease groups in MultiArray1, MultiArray2, and GSE83148, with statistical significance assessed by the Wilcoxon rank-sum test (P < 0.05).

### Diagnostic evaluation of candidate stem-related genes

For validating our findings and diagnostic accuracy of candidate stem-related genes, receiver operating characteristic (ROC) curves were constructed and the area under the curve (AUC) was calculated using “pROC” package. An AUC value > 0.5 was considered diagnostic significantly. Furthermore, multivariable logistic regression analysis using “logistf” package was employed to develop a multi-gene diagnostic model. The overlapping genes *SSR2* and *UBE2D3* evaluation between single-gene and multi-gene diagnostic models was conducted by parallel ROC curve analysis. To obtain a robust estimate of the combined diagnostic model's performance and mitigate overfitting, bootstrap validation with 1,000 iterations was performed on each dataset (MultiArray1, MultiArray2, and GSE83148). The 95% confidence intervals for the AUC were derived from the 2.5th and 97.5th percentiles of the bootstrap distribution.

### Survival analysis

HBV-HCC samples datasets with clinical data and survival data were downloaded from TCGA-LIHC-HBV(+). The survival analysis of target genes was performed by using “survival” package (https://CRAN.R-project.org/package=survival) stratified by median *SSR2* and *UBE2D3* expression into high and low groups.

### Functional correlation analysis

To investigate the potential mechanistic roles of *SSR2* and *UBE2D3* beyond their clinical utility, we performed gene set enrichment and correlation analysis. Gene sets representing key biological processes were curated from MSigDB (https://www.gsea-msigdb.org/gsea/msigdb/index.jsp), including HBV infection signature (“WP_HEPATITIS_B_INFECTION”), the mTOR pathway signature (“BIOCARTA_MTOR_PATHWAY”), the ERS signature (“GOBP_RESPONSE_TO_ENDOPLASMIC_RETICULUM_STRESS”), stemness signatures (“BENPORATH_SOX2_TARGETS” and “BENPORATH_OCT4_TARGETS”). Using the GSVA method, enrichment scores for each of these pathways were calculated for the TCGA-LIHC-HBV+ cohort. Subsequently, Spearman correlation analysis was conducted between the expression levels of *SSR2* and *UBE2D3* in TCGA-LIHC-HBV+ cohort and the corresponding GSVA activity scores for each pathway to assess their functional co-variation.

## Results

### Cells clustering and endothelial subtypes characterization in HCC tissues

To identify specific cell populations with potential CSC properties, thereby facilitating the investigation of biological markers associated with this CSC subpopulation, we analyzed scRNA-seq data from three paired HBV-positive and HBV-negative HCC tissue samples (GEO accession: GSE282701). After quality control, 65,357 high-quality cells were retained for downstream analysis. UMAP of the integrated PCA space revealed 11 transcriptionally distinct cell clusters.

**Fig 2:**
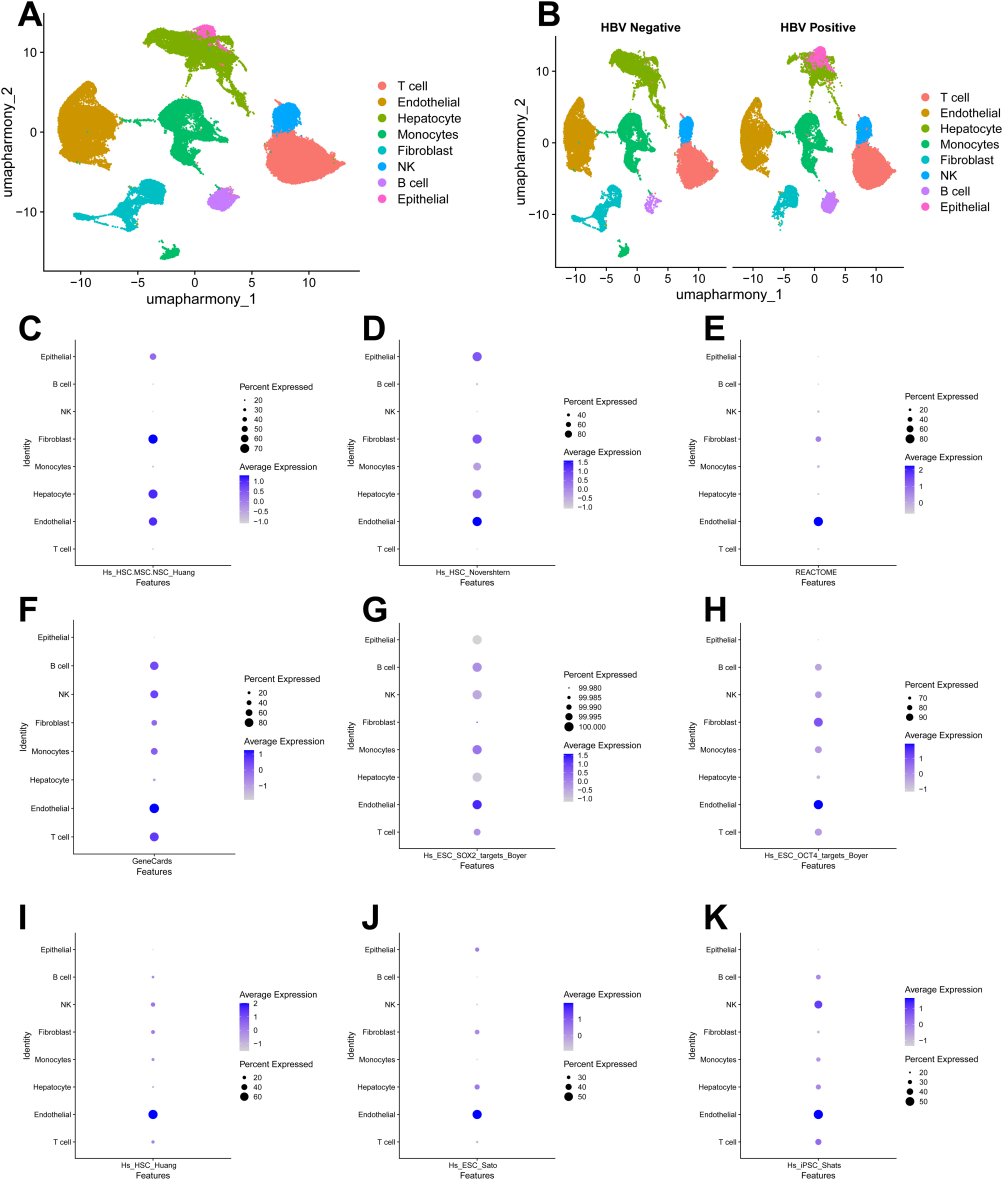
Cell clustering and prediction of stem cell cluster. (A) 11 cell clusters are annotated into eight cell types. (B) UMAP plot showed the different cellular distribution between HBV-positive and HBV-negative. (C-K) Dotplot showed the enrichment scores for 9 established stemness-associated gene sets were calculated across major cell types. *GSE282701 (3 HBV-positive HCC samples and 3 HBV-negative HCC samples) was used in this analysis.

Cell types were annotated by identifying the top five differentially expressed genes per cluster, which were cross-referenced with the Cell Marker and PanglaoDB databases, supplemented by expression quantification of canonical lineage markers. These approaches defined eight major cell populations: T cells (16,674), Endothelial cells (15,522), Hepatocytes (11,262), Monocytes (10,237), Fibroblasts (4,898), NK cells (3,226), B cells (2,138), and Epithelial cells (1,400). We subsequently compared the cellular distributions between the HBV-positive and HBV-negative cohorts (Figs 2A and 2B).

HCC is characterized by pronounced vascularization and vascular heterogeneity. During precancerous lesions, HCC’s aberrant activation of endothelial cells establishes a pro-tumorigenic microenvironment through functional and structural anomalies. The proliferation of blood vessels provides essential nutrients and structural support for tumor growth, while also contributing to the establishment of an immune-privileged environment. Notably, within the inflammatory tumor microenvironment (TME), endothelial cells promote the acquisition of stemness in cancer cells through sustained secretion of pro-inflammatory cytokines. Conversely, subsets of HCC-CSCs reciprocally promote tumor angiogenesis, for instance, by secreting IL-8 to activate the IL-8–ERK signaling cascade [25,26]. Therefore, this study primarily focuses on the role of tumor-associated endothelial cells (TECs) within the TME and their potential bidirectional differentiation with CSCs during HCC progression [27,28]. To objectively evaluate the stemness landscape, we performed a systematic stemness potential assessment for all major cell types based on a compendium of 9 established stemness-associated gene sets. This comparative analysis revealed that endothelial cells consistently exhibited the highest stemness enrichment scores across all gene sets, particularly in the “Hs_HSC_Novershtern”, “Hs_iPSC_Shats”, “Hs_ESC_OCT4_targets_Boyer”, “Hs_ESC_Sato GeneCards”, “Hs_ESC_Sato”, “Hs_ESC_SOX2_targets_Boyer”, “Hs_HSC.MSC.NSC_Huang”, “Hs_HSC_Huang”, “GeneCards”, “REACTOME” (Figs 2C-2K). Consequently, we selected endothelial cells for subsequent analysis. Immune-related cell subtypes were excluded due to their lack of potential for differentiation into CSCs. The remaining stroma-associated cell subtypes, although not the focus of this study, will be investigated in future research to further elucidate the malignant biological features of HCC.

### Identification of a stem-like endothelial progenitor subpopulation (Cluster 4) in HCC

**Fig 3:**
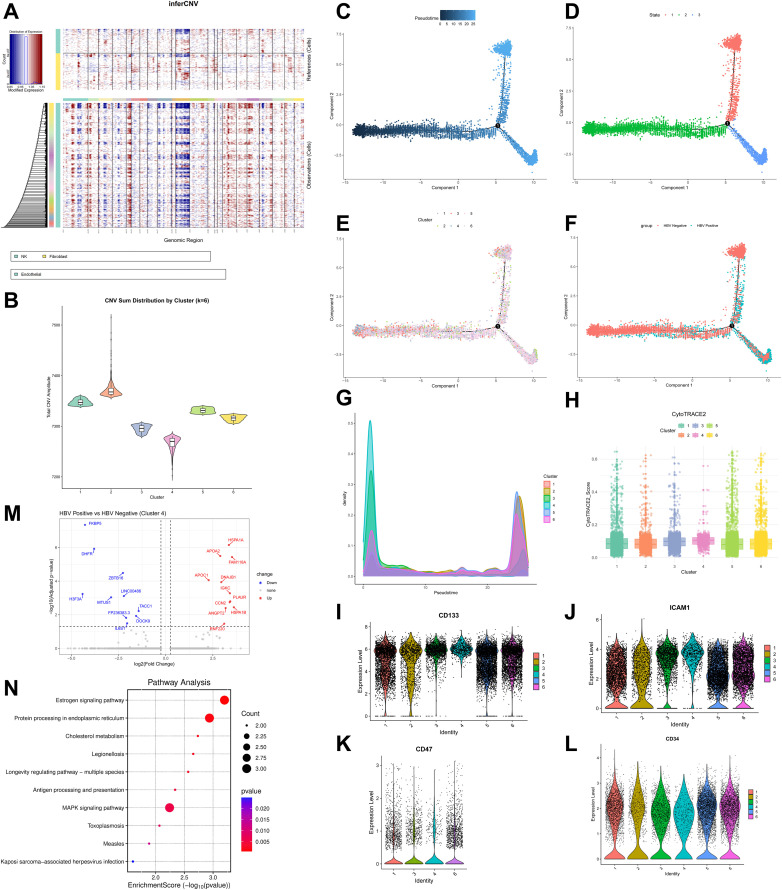
Screening differentially positive-HBV-expressed genes (DPEGs) using scRNA-seq data. (A) Copy number variation (CNV) analysis in single-cell transcriptomic data. In the reference panel, green denotes NK cells and yellow represents fibroblasts; in the observed data, green indicates endothelial cells; in the plot, blue signifies DNA copy number loss, while red corresponds to DNA copy number amplification. (B) CNV score after K-means clustering. (C-F) The Monocle2 analysis of the differentiation trajectory of endothelial cells, presented as pseudotime, state, cluster, and group. (G) The distribution of cluster 1–6 on the pseudotime. (H) The CytoTRACE2 analysis evaluated the stem potential among cluster 1–6. (I-L) Violin plots for CD47, ICAM1, CD133, CD34 expression in clusters 1–6 (if they have expression). (M) Volcano plot visualized the differentially expressed genes between HBV (+) and HBV (-) HCC tissues in cluster 4. (N) KEGG pathway analysis of differentially expressed genes in Cluster 4 between HBV-positive and HBV-negative samples (significance threshold: P-value < 0.05). *GSE282701 (3 HBV-positive HCC samples and 3 HBV-negative HCC samples) was used in this analysis.

Based on the established association between endothelial cells and CSCs, we focused subsequent analyses on the endothelial cell population to identify CSC subpopulations. To delineate heterogeneity within the endothelial population, we performed CNV analysis, which partitioned the cells into six distinct subclusters based on CNV amplitude (Figs 3A and 3B). Our analytical approach was guided by the biological rationale that quiescent CSCs, due to their reduced proliferative activity, accumulate fewer replication errors and thus display lower CNV burdens. Conversely, malignant endothelial cells demonstrate elevated CNV frequencies attributable to persistent exposure to microenvironmental stresses and increased DNA replication error rates during frequent cell divisions in tumor progression. Consequently, we hypothesized that the subcluster with the lowest CNV score would represent a CSC-enriched population.

Based on the premise that CSCs function as tumor-initiating cells (TICs), we next applied pseudotemporal trajectory analysis to order the endothelial cells along a differentiation trajectory. This analysis aimed to identify clusters enriched at the origin of the trajectory, which are postulated to possess the most primitive, CSC-like state (Figs 3C-3F). Cluster 4 was predominantly localized at the origin of the pseudotime axis (Fig 3G), suggesting primitive cellular states. Furthermore, independent computational assessment of stemness potential using the CytoTRACE2 algorithm suggested that Cluster 4 exhibited the highest stemness scores among all endothelial subclusters (Fig 3H).

Furthermore, we evaluated classical markers for validation, including the HCC-CSC markers CD47, ICAM1, CD133, and the mature endothelial marker CD34 (Fig 3I-3L). The elevated CD47, ICAM1, CD133, reduced CD34, and high CytoTRACE scores collectively suggest that Cluster 4 represents a distinct, non-classical stem-like state within the tumor endothelium.

Within the putative stemness-associated Cluster 4, we identified Differentially Positive-HBV-Expressed Genes (DPEGs) by comparing HBV-positive and HBV-negative samples. This analysis yielded 21 HBV-associated candidate genes, comprising 10 downregulated and 11 upregulated genes (Fig 3M). KEGG pathway enrichment analysis of these DPEGs demonstrated significant involvement in four key pathways (Fig 3N): (1) Protein processing in endoplasmic reticulum (ER): likely attributable to ERS induced by viral protein synthesis, a process that sustains stemness and activates pro-angiogenic factors [29]. (2) MAPK signaling: potentially through HBx-induced Ras/Raf/MAPKK/MAPK cascade activation that promotes AP-1-dependent proliferation [30]. The coordinated enrichment of these pathways constitutes an “oncogenic-immunosuppressive axis” that may sustain the equilibrium phase of cancer immunoediting [31]. This pattern suggests that HBV co-opts host stemness regulatory networks to establish a tripartite virus-stem cell-immunity interplay, fostering a malignancy-permissive microenvironment.

### Integrated stemness signature enrichment and WGCNA prioritize stemness-associated hub genes

**Fig 4:**
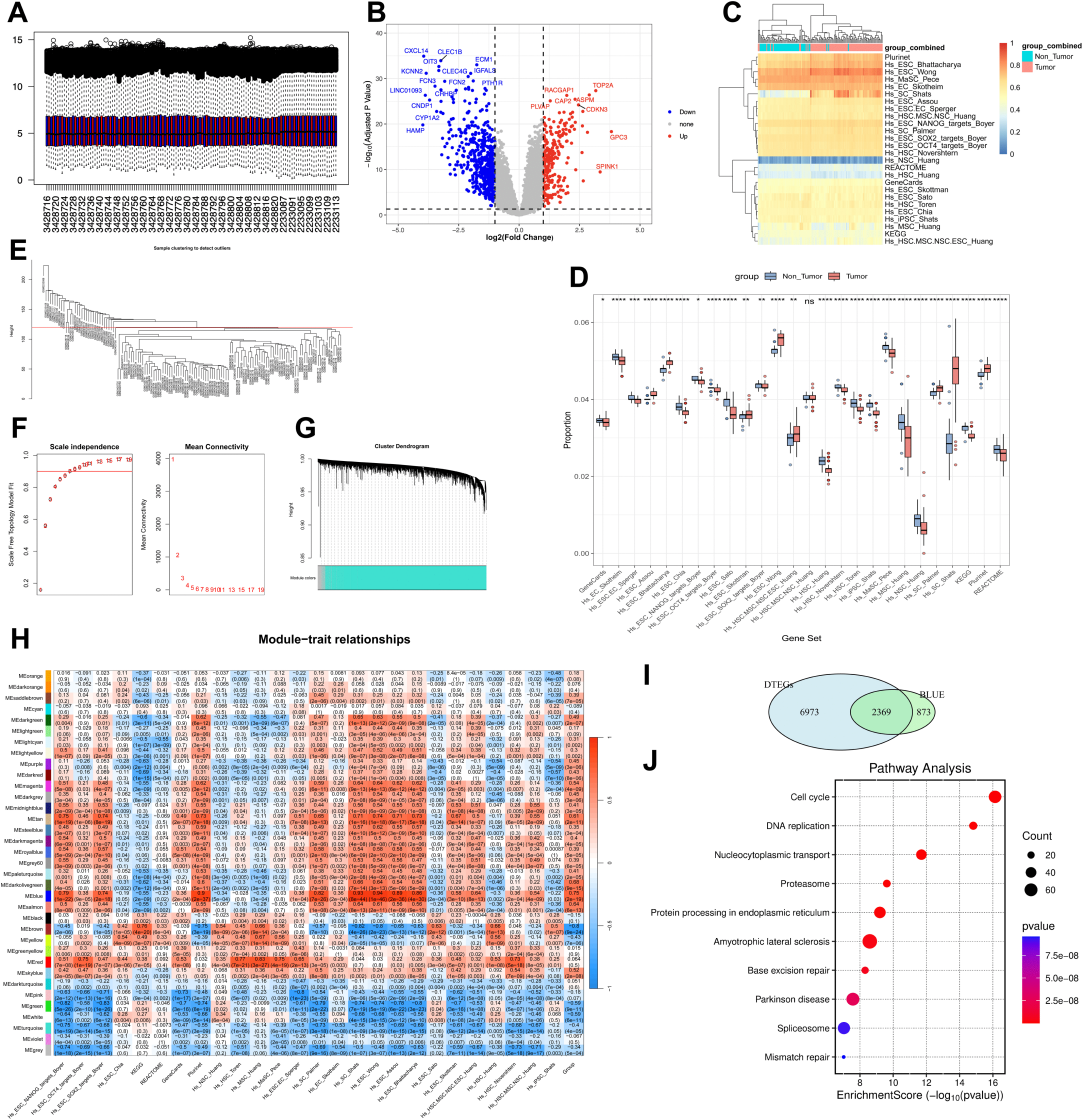
Screening tumor-associated stemness genes (DEGs) using bulk RNA-seq data. (A) Normalization of the MultiArray1. (B) Volcano plot visualizes the differentially expressed genes between tumor and non-tumor tissues. (C) Heatmap illustrates the landscape of ssGSEA stemness scores in tumor and non-tumor tissues. (D) Box plot displays the differences of 26 ssGSEA stemness scores in tumor and non-tumor tissues. ***P < 0.001. (E) Exclude the outlier data where h is greater than 120. (F) Multiple soft thresholding parameters (β) ranging from 1 to 20 in terms of scale independence and mean connectivity. (G) A clustered dendrogram constructed from weighted correlation coefficients, where genes with similar expression patterns are clustered into co-expression modules, each represented by a different color. (H) Heatmap depicts the correlation between module eigengenes (ME) and tumor status as well as stem cell subtypes. (I) Venn plot of differentially expressed genes from DTEGs and marker genes in MEblue. (J) KEGG for the intersection of 2,369 genes (significance threshold: P-value < 0.05). *MultiArray1(GSE121248 and GSE84402; 83 HBV-positive HCC samples and 50 HBV-positive adjacent non-tumorous liver tissues) was used in this analysis.

To complement the single-cell analysis and identify a more comprehensive set of CSC-related genes, we leveraged bulk RNA-seq data. We analyzed the MultiArray1 dataset (GSE121248, GSE84402), comprising 107 tumor and 50 adjacent non-tumor liver tissues from HBV-infected patients (Fig 4A). First, we analyzed differentially expressed tumor-enriched genes (DTEGs), revealing 9,099 downregulated and 243 upregulated DTEGs in HCC compared to adjacent tissues (Fig 4B).

To specifically interrogate stemness properties, we then performed ssGSEA using 26 established stemness signatures (Figs 4C and 4D). Although minimal enrichment was observed in Hs_NSC_Huang and Hs_HSC_Huang sets, tumor tissues exhibited broad activation across most stemness signatures, indicating enhanced stemness-related programs.

Leveraging ssGSEA-derived stemness enrichment scores, we next performed WGCNA on 101 samples after excluding 32 outliers (Fig 4E). A soft-thresholding power (β) of 7 ensured scale-free topology (scale-free fit R² = 0.9) (Fig 4F). Genes were clustered into 35 modules (minimum module size = 30 genes) based on expression similarity (Fig 4G). The results identified a “blue” module that demonstrated the strongest positive correlation with both the tumor phenotype (ME = 0.75, *P* = 2 × 10^−19^) and stemness signatures, particularly Hs_ESC_Wong (ME = 0.94, P = 1 × 10^−46^) and Hs_SC_Shats (ME = 0.93, P = 8 × 10^−44^) (Fig 4H).

The intersection of hub genes from the blue module with DTEGs yielded 2,369 DEGs (Fig 4I). KEGG pathway analysis of these DEGs identified significant enrichment in pathways. Notably, the DNA replication pathway was highly enriched, potentially reflecting HBV-mediated genomic integration and compensatory replication stress response (RSR) mechanisms in CSCs that confer DNA damage tolerance [32]. Additionally, enrichment of the P53 signaling pathway was observed, which may be linked to HBx-induced proteasomal degradation of p53 and subsequent activation of Wnt/β-catenin signaling to sustain CSC self-renewal (Fig 4J) [33].

### Prioritizing biomarkers by integrating functional enrichment and interaction network analysis

**Fig 5:**
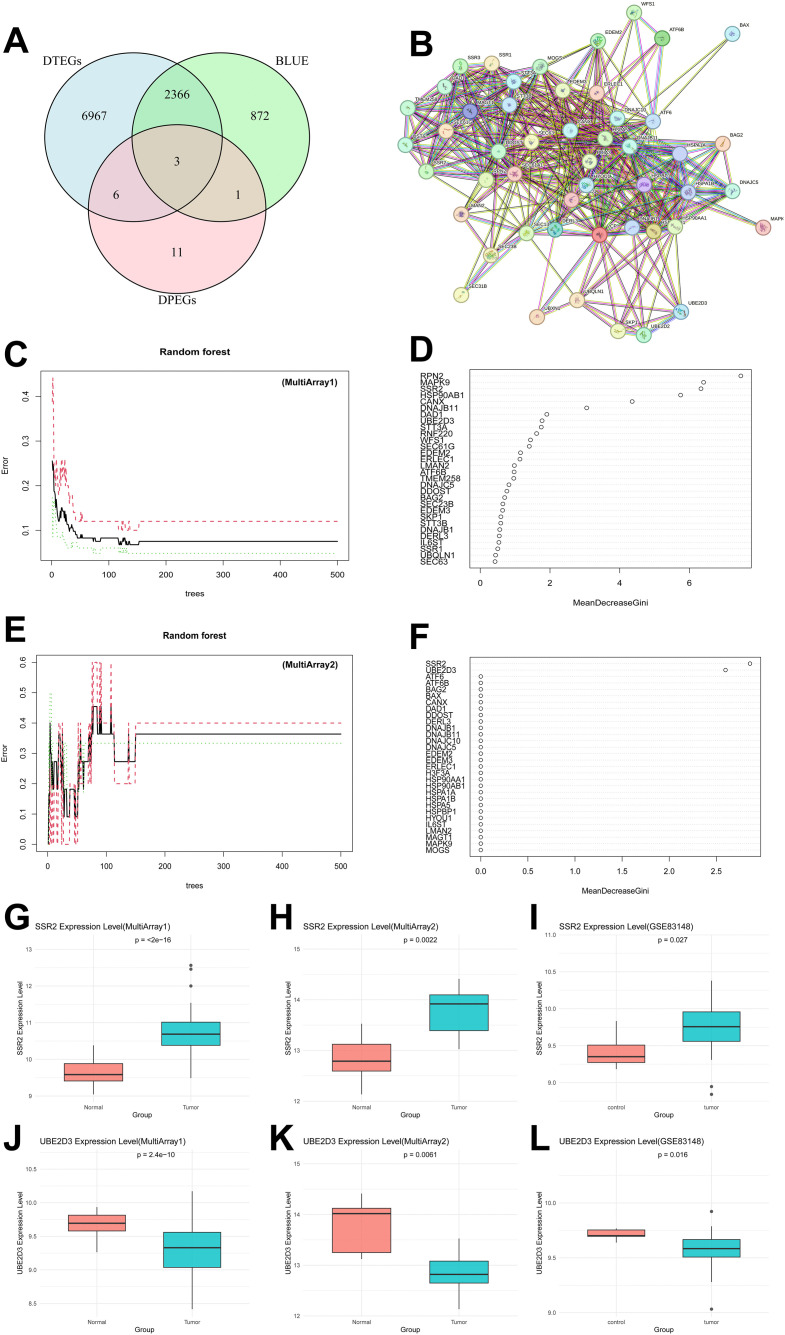
Further screening the hub genes. (A) Venn plot of differentially expressed genes from scRNA-seq and bulk RNA-seq. (B) Protein-protein interaction (PPI) network of genes enriched in the ‘Protein processing in ER’ pathway. (C-D) Analysis based on the random forest algorithm: Relationship between the number of trees and the out-of-bag (OOB) error rate for the HBV(+) HCC versus HBV(+) non-tumorous liver tissue comparison dataset (MultiArray1: GSE121248, GSE84402; 83 HBV-positive HCC samples and 50 HBV-positive adjacent non-tumorous liver tissues), along with the ranking of relative importance scores of genes. (E-F) Analysis based on the random forest algorithm: Relationship between the number of trees and the out-of-bag (OOB) error rate for the HBV(+) HCC versus HBV(-) HCC comparison dataset (MultiArray2: GSE272035, GSE104310; 10 HBV-positive HCC and 15 HBV-negative HCC), along with the ranking of relative importance scores of genes. (G-L) Expression levels of *SSR2* and *UBE2D3* in the MultiArray1 (GSE121248 and GSE84402; 83 HBV-positive HCC samples and 50 HBV-positive adjacent non-tumorous liver tissues), MultiArray2 (GSE272035 and GSE104310; 10 HBV-positive HCC and 15 HBV-negative HCC) and GSE83148 (122 HBV-positive HCC tissues and 6 HBV-negative normal liver tissues).

To refine the list of candidate genes from multi-omics screening, we first identified the intersection of DPEGs and DEGs. They were *RNF220, IL6ST*, and *H3F3A* (Fig 5A). Among these, *IL6ST* (gp130), which is enriched in viral carcinogenesis pathways, functions as a signal-transducing subunit for interleukin-6 family cytokines (*IL-6, LIF, CNTF* and *CTF1*), orchestrating immune regulation, hematopoiesis, and neural survival. In HCC, IL6ST accumulates due to downregulated chaperone-mediated autophagy (CMA) receptor LAMP2A, serving as a KFERQ motif-bearing CMA substrate that promotes tumor proliferation and migration when CMA is impaired [34].

KEGG pathway analysis revealed significant co-enrichment of both gene sets in the “Protein processing in endoplasmic reticulum” pathway. HBV-S protein competitively binds Reticulon 3 (RTN3), coupled with the low expression of RTN3 in HCC, inhibiting the Chk2/p53 signaling axis and impairing apoptosis [35]. HBx, in the meantime, triggers ERS-dependent apoptosis via the CREB3L3-JUN-TP53-CDKN1A cascade [36]. ERS interacts with autophagy to promote HBV replication and secretion [37,38]. Concurrently, ERS activates the CREB5 super-enhancer (CREB5-SE) mediated upregulation of Tenascin-C (TNC) protein, remodeling the extracellular matrix (ECM) and enhancing the invasive capacity of CSCs [39]. Furthermore, SCD1-mediated lipid desaturation attenuates ERS signaling, leading to downregulation of AMP-dependent transcription factor (ATF3) expression. This consequently relieves the transcriptional repression of the CSC marker oncogene v-myc avian myelocytomatosis viral oncogene neuroblastoma derived homolog (MYCN) [40].

PPI network constructed from 49 key genes in this pathway (Fig 5B) identified critical functional modules: (1) Stemness Maintenance Module: the module included ribosome-binding protein RPN2, which stabilizes snail and mutant p53 (mtp53) to enhance the epithelial-mesenchymal transition (EMT) process [41]. (2) Immune Evasion Module: STT3A and STT3B promote PD-L1 glycosylation and stability, and guanine monophosphate synthase (GMPS) enhances this process in HCC, leading to inhibition of CD8 ⁺ T cell function [42,43]. (3) Immunometabolic Regulation Module:Magnesium transporter MAGT1 maintains NKG2D expression critical for NK/CTL cytotoxicity, and its deficiency may contribute to impaired HBV control and tumorigenesis [44,45]. In CSCs, MAGT1 knockout induces S-phase arrest and apoptosis via the ERK/p38-p21/MYC axis, suggesting its potential as a stemness regulatory target [46].

Collectively, our analysis indicated that the ERS pathway exhibits a “pro-apoptotic and pro-survival” dual role in HBV-HCC. Its intricate interaction network coordinates stemness maintenance, immune evasion, and metabolic reprogramming, establishing the TME framework that supports persistent viral infection and tumor progression.

Following the initial screening of 52 genes, we employed RF machine learning algorithm to refine potential biomarkers. Two distinct datasets were utilized for model training and evaluation: MultiArray1 (HCC Histological Status), comprising gene expression profiles comparing tumor tissue versus adjacent non-tumorous liver tissue (GEO accessions: GSE121248, GSE84402); MultiArray2 (HBV Infection Status), comprising gene expression profiles comparing HBV-positive versus HBV-negative HCC samples (GEO accessions: GSE272035, GSE104310).

Application of RF to MultiArray1 identified nine genes (*RPN2, MAPK9, SSR2, HSP90AB1, CANX, DNAJB11, DAD1, UBE2D3, SST3A*) with relative feature importance score exceeding 2 (Figs 5C and 5D). Similarly, analysis of MultiArray2 identified two genes (*SSR2, UBE2D3*) with a relative feature importance score > 1.5 (Figs 5E and 5F). The overlapping genes *SSR2* and *UBE2D3* from both models were selected as candidate diagnostic biomarkers for further validation.

Then, we assessed the expression level of *SSR2* and *UBE2D3*. The results showed that *SSR2* expression was significantly upregulated, while *UBE2D3* was significantly downregulated in HBV-positive HCC tissues compared to control groups (all p < 0.05, Wilcoxon test) in both discovery cohorts (HCC Histological Status cohort MultiArray1 and HBV Infection Status cohort MultiArray2) (Figs 5G-5L). This consistent dysregulation pattern was successfully replicated in the independent validation cohort GSE83148 (p < 0.05), thereby strengthening the evidence for their association with HBV-HCC.

### *SSR2* and *UBE2D3* are the potential stem-related genes with clinical relevance in HBV-HCC

To validate the clinical relevance of *SSR2* and *UBE2D3*, we assessed their diagnostic and prognostic value in HBV-HCC.

**Fig 6:**
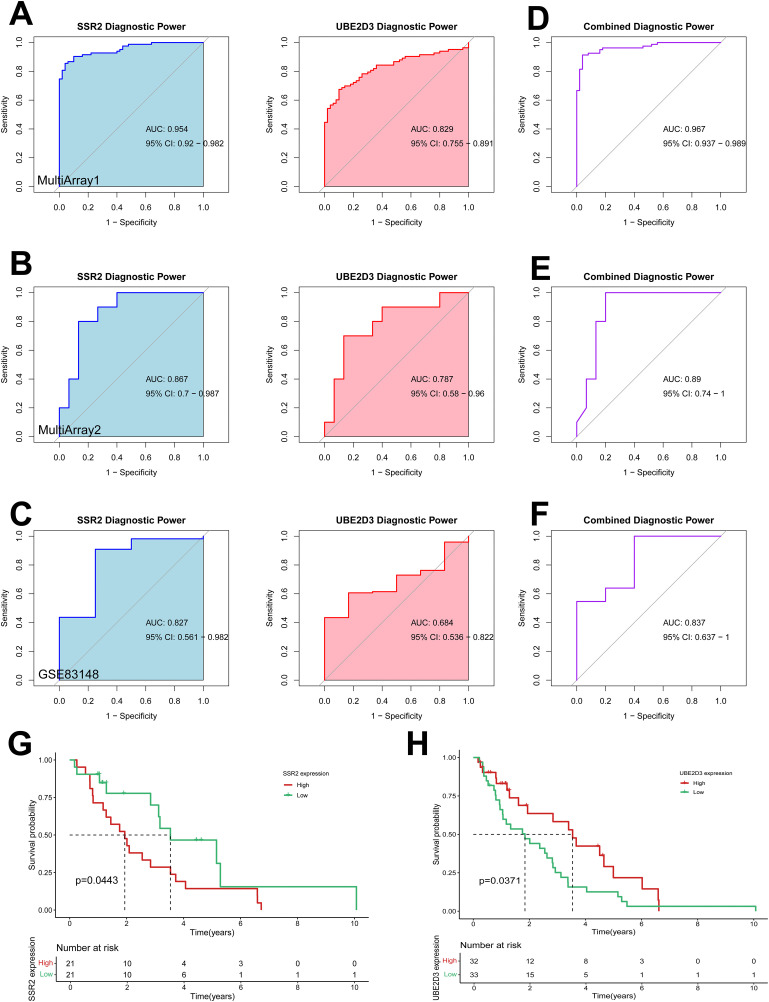
Diagnostic and prognostic value of *SSR2* and *UBE2D3* in HBV(+) HCC patients. **(A-C)** Receiver operating characteristic (ROC) curve of the two shared genes (*SSR2* and *UBE2D3*) in test (MultiArray1, 83 HBV-positive HCC samples and 50 HBV-positive adjacent non-tumorous liver tissues; MultiArray2, 10 HBV-positive HCC and 15 HBV-negative HCC) and validation (GSE83148, 122 HBV-positive HCC tissues and 6 HBV-negative normal liver tissues) datasets for HBV and HCC. (D-F) ROC curve of the multi-marker diagnostic model in test (MultiArray1, 83 HBV-positive HCC samples and 50 HBV-positive adjacent non-tumorous liver tissues; MultiArray2, 10 HBV-positive HCC and 15 HBV-negative HCC) and validation (GSE83148, 122 HBV-positive HCC tissues and 6 HBV-negative normal liver tissues) datasets for HBV and HCC. (G) Kaplan–Meier survival analysis of HBV-HCC patients stratified by median *SSR2* expression into high and low groups using TCGA-LIHC-HBV(+) database (105 HBV-positive HCC patients). (H) Kaplan–Meier survival analysis of HCC patients stratified by median *UBE2D3* expression into high and low groups using TCGA-LIHC-HBV(+) database (105 HBV-positive HCC patients).

ROC analysis was conducted in discovery cohorts MultiArray1and MultiArray2, and validation cohort GSE83148 separately. The results showed that *SSR2* and *UBE2D3* demonstrated significant diagnostic potential in both comparisons, with AUC values exceeding 0.5 (Figs 6A-6C). To improve disease prediction, we utilized logistic regression to integrate the two hub genes and develop a multi-marker diagnostic model. The combined model achieved superior diagnostic accuracy compared to either gene alone, with AUC values of 0.967 (95% CI: 0.937–0.989) in dataset MultiArray1, 0.890 (95% CI: 0.740–1.000) in dataset MultiArray2 and 0.837 (95% CI: 0.637–1.000) in GSE83148 (Figs 6D-6F). These findings demonstrate that the *SSR2/UBE2D3* model exhibits excellent predictive power for identifying HBV-HCC patients.

We next evaluated the prognostic significance of *SSR2* and *UBE2D3* expression using clinical data from TCGA-LIHC-HBV(+). Patients were stratified into high- and low-expression groups based on median gene expression levels. Kaplan-Meier survival analysis revealed strong, statistically significant associations between gene expression levels and overall survival (OS) in HBV-HCC patients.

Consistent with its upregulation in HBV-HCC, higher expression levels of *SSR2* were significantly associated with reduced OS (Fig 6G). Conversely, consistent with its downregulation in HBV-HCC, higher expression levels of *UBE2D3* were significantly associated with prolonged OS (Fig 6H).

Collectively, these analyses establish the robust diagnostic utility and prognostic relevance of *SSR2* and *UBE2D3* in HBV-HCC.

### Association of *SSR2* and *UBE2D3* with key oncogenic pathway

To bridge the gap between biomarker identification and biological mechanism, we investigated the correlation between the expression of *SSR2* and *UBE2D3* and the activity of key oncogenic pathways in HBV-HCC. Our analysis revealed distinct and significant functional associations for each gene, which can be interpreted through their established biological roles in HBV-HCC.

**Fig 7:**
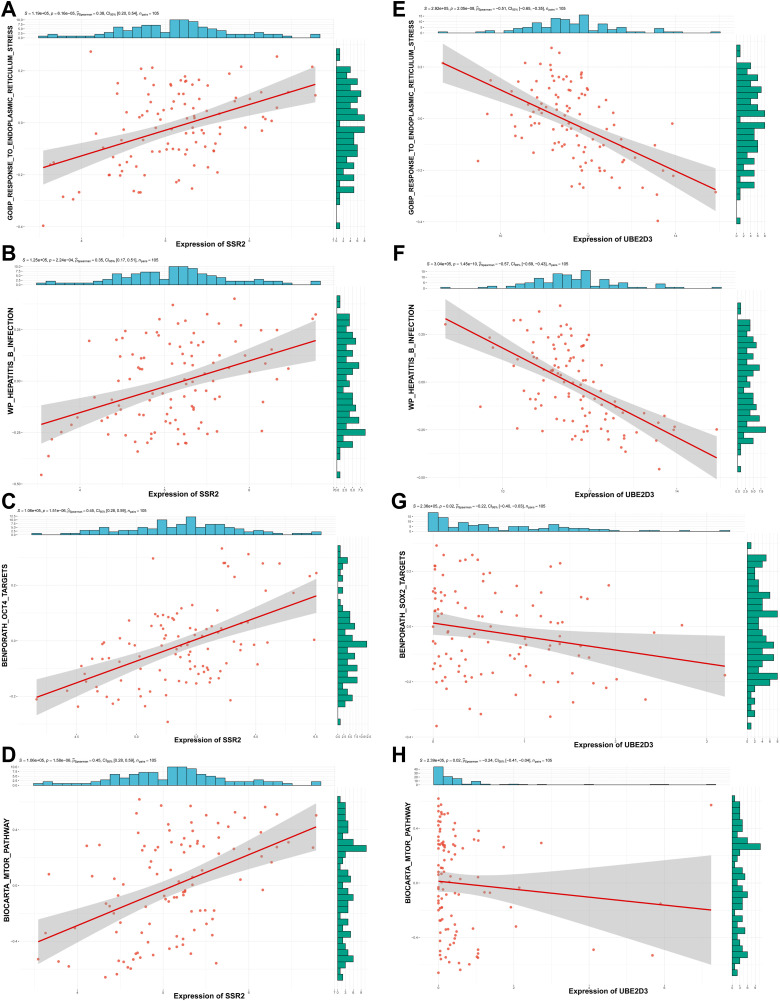
Functional associations of *SSR2* and *UBE2D3* with key oncogenic pathways in HBV-HCC. (A-D) Positive correlation between *SSR2* expression and the HBV infection signature (ρ = 0.35), the endoplasmic reticulum stress (ERS) signature (ρ = 0.38), the OCT4 stemness target set (ρ = 0.45), mTOR signaling activity (ρ = 0.45). (E-H) Negative correlation between UBE2D3 expression and the HBV infection signature (ρ = −0.57), the ERS signature (ρ = −0.51), the *SOX2* stemness target set (ρ = −0.22), mTOR signaling activity (ρ = −0.24). HBV, hepatitis B virus; ERS, endoplasmic reticulum stress. *TCGA-LIHC-HBV(+) (105 HBV-positive HCC patients) database was used in this analysis.

*SSR2* expression showed a strong positive correlation with the HBV infection signature (ρ = 0.35) and with the ERS gene set (ρ = 0.38). These align with its core function as a signal sequence receptor subunit facilitating the co-translational translocation of nascent polypeptides, including viral proteins, into the ER [47]. By enhancing the ER import and processing of HBV and oncogenic proteins, upregulated *SSR2* may directly promote viral persistence and tumorigenesis. Its positive correlation with the OCT4 stemness target set (ρ = 0.45) and mTOR pathway activity (ρ = 0.45) support a model wherein *SSR2* may drive HCC progression by inactivating the Hippo tumor suppressor pathway, while concurrent mTOR activation promotes YAP-dependent transcription by binding to and inducing the degradation of the tumor suppressor ARID1A, thereby upregulating CSC-associated stemness genes (Figs 7A-7D).

*UBE2D3* demonstrated a strong negative correlation with the HBV infection (ρ = −0.57), ERS (ρ = −0.51) signatures, and mTOR pathway activity (ρ = −0.24). As an E2 ubiquitin-conjugating enzyme critical for the ER-associated degradation (ERAD) branch of protein quality control, *UBE2D3* may suppress carcinogenesis by mediating the polyubiquitination and degradation of viral or misfolded proteins via the ubiquitin-proteasome system (UPS), thereby alleviating ERS and limiting viral persistence. Its role in facilitating the repair of HBV-induced DNA damage could further contribute to genomic stability and explain its negative association with the pro-growth mTOR pathway. Its negative correlation with the *SOX2* target set (ρ = −0.22) reinforces its potential tumor-suppressive function, possibly by negatively regulating stemness networks (Figs 7E-7H).

**Fig 8:**
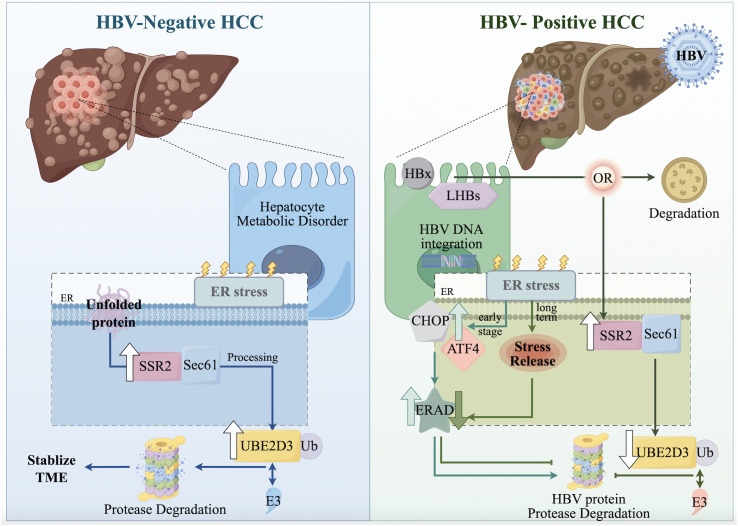
Comparative schematic of ERS mechanisms in HBV-positive and HBV-negative HCC. The left panel (HBV-negative HCC) illustrates that hepatocarcinogenesis is primarily driven by metabolic dysregulation, leading to the accumulation of unfolded proteins and the initiation of ERS. The recruitment of the *SSR2/Sec61* translocon complex facilitates processing and translocation of misfolded proteins for ubiquitin-dependent degradation mediated by *UBE2D3*, which promotes TME stabilization. The right panel (HBV-positive HCC) demonstrates that HBV DNA integration into the host genome, and the expression of viral proteins such as HBx and LHBs, induced ERS. Early-stage responses involve activation of the *CHOP* and *ATF4*, and possible degradation of HBV proteins through ERAD. However, in long-term infection, *UBE2D3* is suppressed, impairing ERAD function, alleviating ER stress, and enabling persistent HBV protein expression and tumor progression.

**Fig 9:**
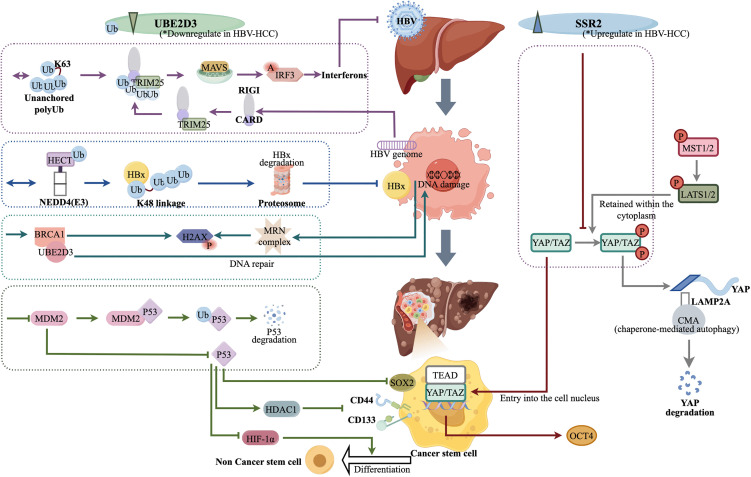
The divergent roles of *UBE2D3* and *SSR2* in HBV-HCC. *UBE2D3*, downregulated in HBV-HCC, acts as a tumor suppressor and antiviral factor via multiple mechanisms: (1) It complexes with an E3 ubiquitin ligase to mediate K48-linked polyubiquitination (polyUb) of the viral HBx protein, targeting it for proteasomal degradation; (2) It catalyzes the formation of unanchored K63-linked polyUb chains, activating antiviral immunity; (3) It suppresses tumorigenesis by stabilizing the tumor suppressor p53 (inhibiting MDM2-mediated degradation), thereby promoting cancer cell differentiation and suppressing stemness; and (4) It forms a complex with BRCA1 to facilitate repair of HBV-induced DNA damage. Conversely, *SSR2*, upregulated in HBV-HCC, promotes oncogenesis by inactivating the Hippo pathway: *SSR2* prevents phosphorylation of YAP/TAZ transcription factors, resulting in their release from cytoplasmic retention and subsequent translocation to the nucleus, where they initiate translation of CSC markers. Arrows denote molecular pathways and interactions; specific colors represent distinct biological processes as shown in the schematic.

These opposing correlation patterns underscore that *SSR2* and *UBE2D3* are functionally linked to the dysregulation of specific pathways critical for viral persistence, cellular stress, stemness, and metabolism in HBV-HCC. During HBV-driven oncogenesis, upregulated *SSR2* likely enhances the ER import and processing of viral proteins, while the downregulation of *UBE2D3* impairs ERAD-mediated clearance of these proteins and compromises DNA damage repair. This dual dysregulation of the protein quality control system creates a permissive environment for viral persistence, proteotoxic stress, and subsequent tumor progression, solidifying their roles as potential regulators in pathogenesis (Figs 8 and 9).

## Discussion

To address the limited research specifically investigating the impact of HBV on CSCs within the context of HCC onset, our study systematically explored the functional mechanisms of CSC signature genes, namely *SSR2* and *UBE2D3*, in HBV-HCC by integrating scRNA-seq and bulk RNA-seq analyses.

Our findings identify *UBE2D3* as downregulated in HBV-HCC. Many studies have demonstrated its role in catalyzing ‘Lys-48’-linked polyubiquitination for ERAD-mediated proteasomal degradation of misfolded proteins [48], modulating the p53 pathway by facilitating both MDM2-mediated p53 ubiquitination (which reduces p53 stability) and MDM2 autoubiquitination (which stabilizes MDM2 and sustains p53 repression), leading to an accumulation of transcriptionally inactive p53 [49]. *UBE2D3* also contributes to antiviral immunity by cooperating with E3 ligases to generate ‘Lys-63’-linked polyubiquitin chains that activate the MAVS-RIG-I signaling pathway [50]. While direct evidence linking *UBE2D3* downregulation specifically to HBV-HCC remains limited, its role is strongly supported by parallel dysregulation of ubiquitination pathways in HBV-associated hepatocarcinogenesis. Ubiquitin-proteasome pathway-associated pseudogene UPAT depletion impairs ZEB1 degradation, promoting EMT and CSC properties [51]. HECT family E3 ligase NEDD4-mediated ‘Lys-48’-polyubiquitination and degradation of HBx exert tumor-suppressive effects [52]. TRIM29-dependent ubiquitination of hyaluronic acid-mediated motility receptor (HMMR) induced by chronic infection alleviates ERS to delay HCC progression [53]. Collectively, these findings from related ubiquitination pathways indirectly substantiate the protective role of *UBE2D3* in counteracting the chronic progression of HBV-HCC.

In contrast to *UBE2D3*, *SSR2* is upregulated in HBV-HCC and is associated with poor prognosis. *SSR2* may promote tumorigenesis by inactivating the tumor-suppressive pathways—Hippo. The oncogenic potential of the Hippo pathway effectors YAP/TAZ is dependent on their dephosphorylation, nuclear translocation, and interaction with TEAD family transcription factors. Research indicates that *SSR2* may prevent YAP/TAZ phosphorylation, facilitating their nuclear accumulation. Once in the nucleus, YAP/TAZ-TEAD complexes activate the transcription of a suite of pro-oncogenic target genes, including key CSC markers (e.g., *SOX9*, *OCT4*) and EMT-related genes, which collectively enhance the metastatic potential and invasive capacity of CSCs [54,55].

Although our analysis did not find evidence of a direct molecular interaction between *SSR2* and *UBE2D3*, KEGG placed both genes within the broader context of ER protein processing pathways [56]. This commonality is highly significant, given the well-established and multifaceted role of ERS in the pathogenesis of both chronic HBV infection and HCC [57,58]. Notably, ERS exhibits a dual role in HBV infection. For example, viral replication and the accumulation of viral proteins HBx induce ERS, activating unfolded protein response (UPR), which can promote HBV replication and disease progression [59]. Conversely, ERS triggered by factors such as HBx and excess HBsAg can disrupt calcium homeostasis, leading to the induction of IL-8 transcription and contributing to anti-viral responses [60]. Similarly, ERS has a dualistic impact in cancer: tumor cells exploit ERS to manage proteotoxic stress and support rapid growth, aiding in maintaining cellular homeostasis, while persistent or severe ERS can ultimately trigger apoptosis [61].

Furthermore, within the chronic HBV infection context, the viral oncoprotein HBx can suppress the expression of the tumor suppressor PTEN, leading to the aberrant activation of the PI3K/AKT/mTOR signaling pathway [14]. Recent studies have extensively highlighted the role of mTOR signaling in LCSC regulation and HBV-HCC progression, where its hyperactivation not only fuels tumor metabolism but also orchestrates immune evasion and stemness maintenance [15]. This activation subsequently induces the phosphorylation of STAT3, which in turn upregulates a series of stemness-related genes, thereby promoting the acquisition and maintenance of CSC properties. Complementing this viral trigger, intrinsic oncogenic events in HCC, such as the overexpression of the small GTPase Rab1A, further promote amino acid-dependent hyperactivation of mTORC1 [16]. This convergent overactivation of mTOR signaling exerts profound oncogenic effects. Additionally, chronic HBV infection and HBx expression induce ERS, which can both influence and be influenced by mTOR signaling. The concurrent dysregulation of the protein quality control system observed in our study likely exacerbates proteotoxic stress [17]. This creates a permissive environment where impaired clearance of viral proteins (due to low *UBE2D3*) and enhanced their processing (due to high *SSR2*) synergize with the HBx-mTOR-driven proliferative and stemness programs, collectively fueling HBV-HCC progression.

Inevitably, there are several limitations in our study. First, as an *in silico* analysis relying on public datasets, our findings lack experimental validation (e.g., Western blot, IHC, or functional assays) to mechanistically verify the roles of *SSR2* and *UBE2D3* in HBV-HCC tissues. The stem-like properties of Cluster 4 are based on computational profiles, and its functional hallmarks remain to be validated experimentally. Furthermore, the role of ERS in HBV-HCC is complex and context-dependent [62], making it challenging to categorically classify *SSR2* or *UBE2D3* as straightforward tumor suppressors or oncogenes. This is underscored by the literature predominantly identifying both genes as risk factors in general HCC [63–65], while our HBV-HCC-specific analysis surprisingly identified *UBE2D3* as a potential protective factor [66]. Future studies integrating epigenomic or proteomic approaches could elucidate the cooperative regulatory networks between HBV integration sites and CSC signature genes, thereby providing a more comprehensive, multi-dimensional understanding of HBV-driven hepatocarcinogenesis.

## Conclusion

In summary, this study employed an integrative multi-omics approach to delineate the transcriptional landscape of HBV-HCC, specifically comparing tumor versus non-tumor tissues and HBV-infected versus non-infected patients. We further explored the diagnostic potential of two candidate stemness-related genes, *SSR2* and *UBE2D3*, and their biological processes in mediating the surveillance and clearance of pro-inflammatory and oncogenic HBV proteins or genes within the ER. Collectively, these findings establish a hypothesis-generating foundation for understanding the molecular mechanisms driving HBV-HCC.
